# Primary intraosseous squamous cell carcinoma arising in dentigerous cyst: Report of 2 cases and review of the literature 

**DOI:** 10.4317/jced.52689

**Published:** 2015-12-01

**Authors:** Cosme Gay-Escoda, Octavi Camps-Font, Marta López-Ramírez, August Vidal-Bel

**Affiliations:** 1 MD, DDS, MS, PhD. Chairman and Professor of Oral and Maxillofacial Surgery. Faculty of Dentistry – University of Barcelona. Director of the Master of Oral Surgery and Implantology (EFHRE International University/FUCSO). Coordinating investigator of the IDIBELL institute. Head of the Department of Oral and Maxillofacial Surgery and Implantology, and Director of the TMJ Disease and Orofacial Pain Unit. Teknon Medical Center. Barcelona, Spain; 2 DDS. Fellow of the Master degree program in Oral Surgery and Implantology. Faculty of Dentistry – University of Barcelona; 3 DDS, MS. Master degree program in Oral Surgery and Implantology. Faculty of Dentistry – University of Barcelona; 4 MD. Specialist in Clinical Pathological Anatomy. Associate Professor of Experimental Clinical Pathology and Terapeutics. School of Dentistry of the University of Barcelona. Researcher of the IDIBELL Institute. Barcelona, Spain

## Abstract

Dentigerous cysts are one of the most common odontogenic cysts of the oral cavity. Odontogenic cysts can give rise to a variety of neoplasms. Carcinoma arising in a dentigerous cyst is extremely rare, with a review of literature showing near 30 cases. The present report describes 2 cases of primary intraosseous squamous cell carcinoma originated from a dentigerous cyst. The first one refers to a 57-year old female with a persistent lesion in the left retromolarregion and diagnosed with squamous cell carcinoma originated fromthe incomplete excision of the lower third molar follicle during its surgical extraction. The second case describes the case of an 18-year old male with an impacted upper canine with previous history of infection and swelling of the oral cavity. The histopathological study revealed the malignization of the follicle surrounding the dental crown. These two cases confirmed the importance of the histopathological study of all the tissue samples obtained from surgical procedures. Although the development of a malignant lesion from a dentigerous cyst is rare and its clinical-radiological features are apparently innocuous, this entity should be considered as a differential diagnosis.

** Key words:**Dentigerous cyst, odontogenic cyst, squamous cell carcinoma, primary intraosseous squamous cell carcinoma, odontogenic carcinoma.

## Introduction

Primary intraosseousodontogenic carcinoma (PIOC) is defined as a squamous cell carcinoma arising within the jawbones; without any initial connection with the oral mucosa or sinus mucosa and develops from remnants of odontogenic epithelium (1).

The term PIOC had been suffered several modifications since was firstly suggested by the World Health Organization (WHO) in 1972 (2). In fact, the use of the term PIOC is considered inappropriate since Eversole *et al.* (3) used the term primary intraosseous squamous cell carcinoma (PIOSCC) to replace it. According to the latest WHO Classification of Tumours classification there are three PIOSCCs subtypes (3):

1. Solid tumour that invades marrow spaces and induces osseous resorption

2. PIOSCC arising from the lining of an odontogenic cyst, making a subdivision in carcinomas arising in a keratocystic odontogenic tumuor (‘keratocyst’) and carcinomas arising in other odontogenic cysts

3. PIOSCC in association with other benign epithelial odontogenic tumours 

The malignant transformation of an odontogenic cyst into a PIOSCCis extremely rare; there are nearly 100 cases in the literature (1). Most of them arise from radicular/residual cysts (60%), although cases originated from dentigerous cysts (16%), keratocystic odontogenic tumour (14%) and lateral periodontal cysts (1%) have been also reported (1,4).

The deﬁnitive diagnosis of PIOSCC is often difficult and usually made in retrospect due to lack of pathognomonic symptoms and radiographic changes. Differential diagnosis includes alveolar carcinomas that have invaded the bone from the overlying soft tissue, from tumours that have metastasized to the jaw from distant sites, from association with some other odontogenictumor, and also from tumors of the maxillary sinus (5).

The aim the present report is to describe the clinical features, therapeutic management and follow-up of two cases of PIOSCC arising indentigerous cysts. The study protocol was approved by the Ethical Committee for Clinical Research (CEIC) of the Dental Hospital of the University of Barcelona.

## Case Report

-Case Report 1

In 1988, a57 year-old female came to the Oral Surgery Unit of the School of Dentistry of the University of Barcelona with anexophytic tumour-like lesion after six-month evolution. Patient’s pathological background comprised stomach, controlled with omeprazole 20 mg orally (1 every 12 hours. (Omapren®, Lesvi, Sant Joan Despí, Spain)). The patient visited her private dental practitioner presenting left submaxillary region swellingrelated to homolateral third molar impaction. The lower second and third left molars were removed, although there is no information regarding the complete excision of the pericoronary lesion as no histopathological examination was carried out. Four weeks after the surgical procedure, patient continued to present slight swelling and trismus. Three moths latter, due to symptoms persistence, curettage and cleaning of the area was carried out without a histopathological examination of the removed tissue.

**Figure 1:**
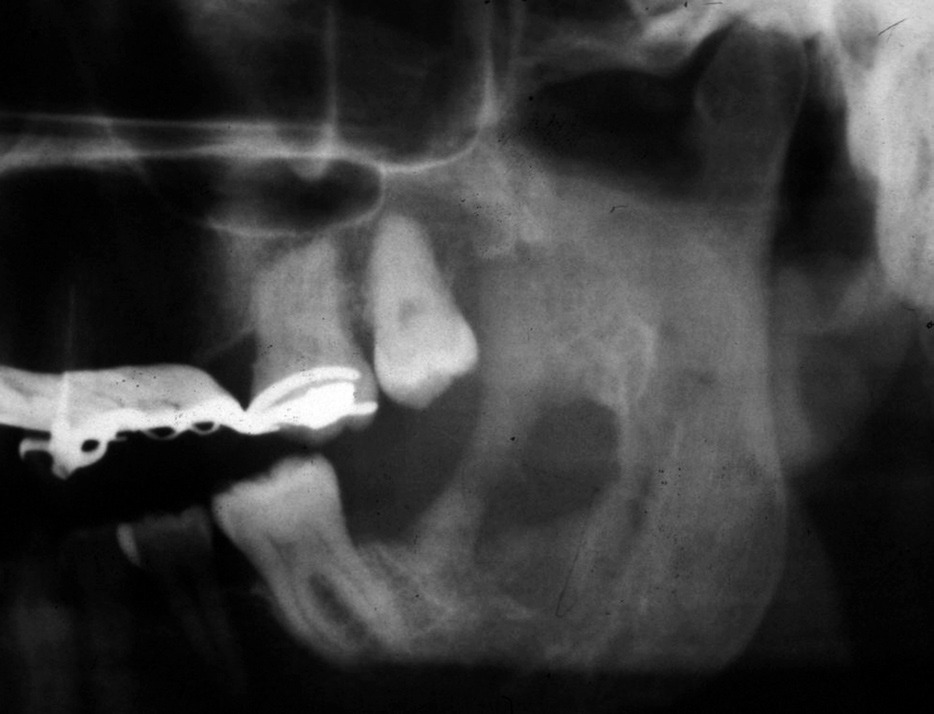
Case report 1. Panoramic X-ray at 4 months after surgical extraction of the lower left second and third molars.

General and regional exploration revealed facial swelling on the left side, slight trismus with neither nerve function impairment nor associated adenopathies. Intraoral examination showed an exophytic tumour-like lesion in there tromolar region. Panoramic X-ray revealed a radiolucent lesion with uneven margins and indents (Fig. 1).

Based on the data obtained, a tentative diagnosis of PIOSCC was established.

**Figure 2:**
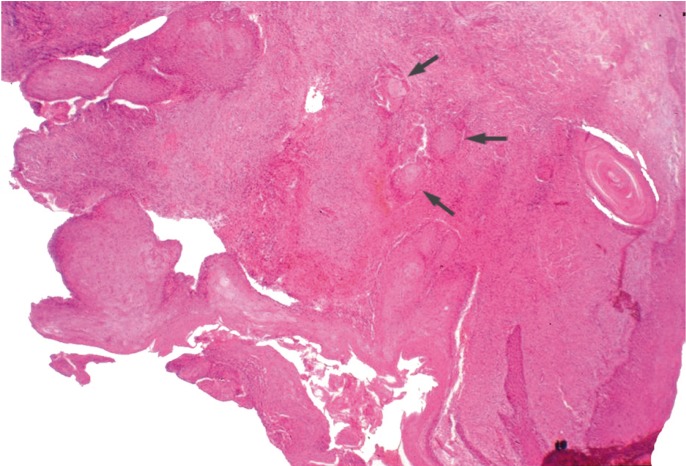
Case report 1. Histological image where squamous cells and cytologicatypia were focally infiltrating the stroma.

Once informed consent was accepted, an incisional biopsy of the osteolytic lesion was performed. The histopathological examination revealed a squamous carcinoma arising in adentigerous cyst. The lesion was covered by stratified squamous epithelium with maturation of keratinocytes and no atypia. Squamous cells and cytologicatypia were noted focally infiltrating the stroma (Fig. 2).

On August 1988, once the Head and Neck Tumour Board reviewed the case, preoperative chemotherapy was administered: one single dose of cistaplin (100 mg/m2) and tegafur (1000 mg/m2/daily) during 2 weeks. Three months later supraomohyoid neck dissection was performed together with hemimandibulectomy to the mental foramen, including the inferior alveolar nerve. The histological study of the cervical lymph nodes, submaxillary glands and the resected tooth showed no malignant invasion.

No postoperative complications were reported. Since then, clinical and radiological follow-up appointments were scheduled every six months, and no evidence of recurrence was found until 1991. As the evolution was favourable, the patient was offered to reconstruct the left posterior region of the mandible using a microvascularized skin engraftment. However the proposed treatment was rejected since the functional and aesthetic sequelae were well tolerated. Follow-up visits have been performed at yearly interval with no recurrence.

-Case report 2

In 2004, an 18 year-old male without pathological backgroundwas referred to the Oral Surgery Unit of the School of Dentistry of the University of Barcelona presenting recurrent painful inflammation of the right canine space. Patient’s dental practitioner performed a surgical debridement of the lesion two weeks before. As purulent drainage and pain were reported, amoxicillin 875 mg and potassium clavulanate 125 mg (p.o every 8 hours for 7 days (Augmentine 875/125; GlaxoSmithKline; Madrid, Spain)) and ibuprofen 600 mg (p.o. every 8 hours for 5 days (Algiasdin®; Esteve, Barcelona, Spain)) were prescribed.

**Figure 3:**
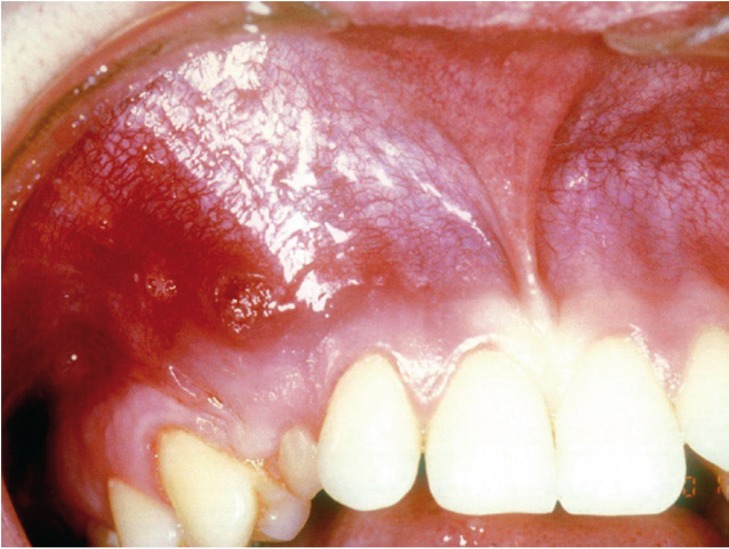
Case report 2. Intraoral examination.

Extraoral and regional examination revealed swelling in the right canine’s fossa, painful to palpation with soft and tender but not fluctuating consistency. Regarding local examination, the oral mucosa showed congestion without ulceration and an active fistulous tract was observed. Furthermore, the presence of the upper temporary canines was also noted (Fig. 3).

**Figure 4:**
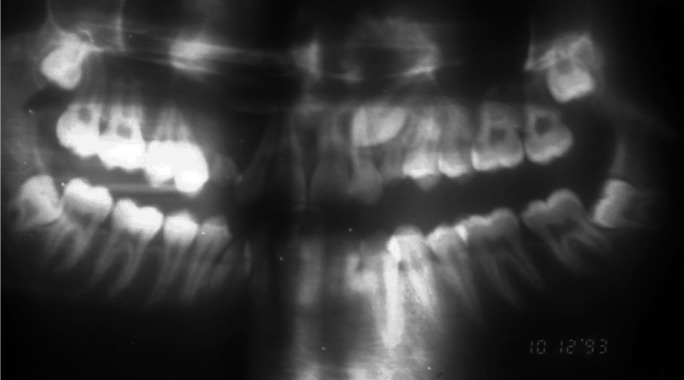
Case report 2. Panoramic X-ray showing the impaction of the upper right canine associated with unilocular radiolucency surrounding the crown.

Panoramic X-ray revealed impaction of both permanent canines with unilocular well-defined radiolucent image surrounding its crowns. Both upper right first premolar and temporary canine showed root resorption, although CO2 vitality tests were un event ful (Fig. 4).

Based on the data obtained, presumptive diagnoses of infected and non-infected dentigerous cysts associated with the upper permanent canines were respectively established.

Once informed consent was accepted, locoregional anaesthesia (articaine in a 4% solution with epinephrine 1:100,000 (Artinibsa®; Inibsa Dental, Lliçà de Vall, Spain)) techniques were employed for the extraction of the upper right temporary and permanent canines and the associated lesion using a buccal approach.

**Figure 5:**
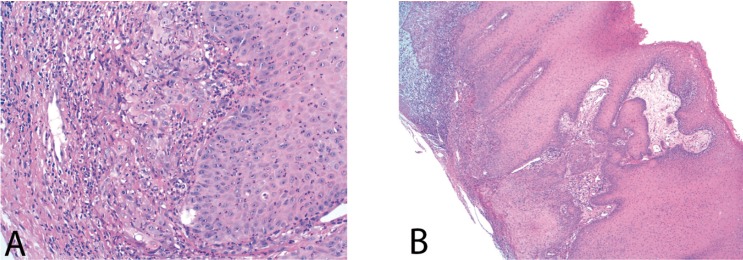
A) Infiltration areas: cytologicatypia was noted focally infiltrating the stroma (HE, x200). B) Cyst wall covered by squamous epithelium showing hyperplasia and infiltrated carcinoma (upper angle) (HE, x40).

Tissue samples obtained were sent to its histopathologic analyses. The results showed a cystic cavity covered by stratified squamous epithelium with marked papillomatosis and acantholysis. In the areas where the acantholytic type was larger, an alteration on keratinocyte maturation and cytologicatypia infiltrating the stroma were observed (Fig. 5). Based on these findings, diagnosis of squamous cell carcinoma a rising in a dentigerous cyst was obtained.

**Figure 6:**
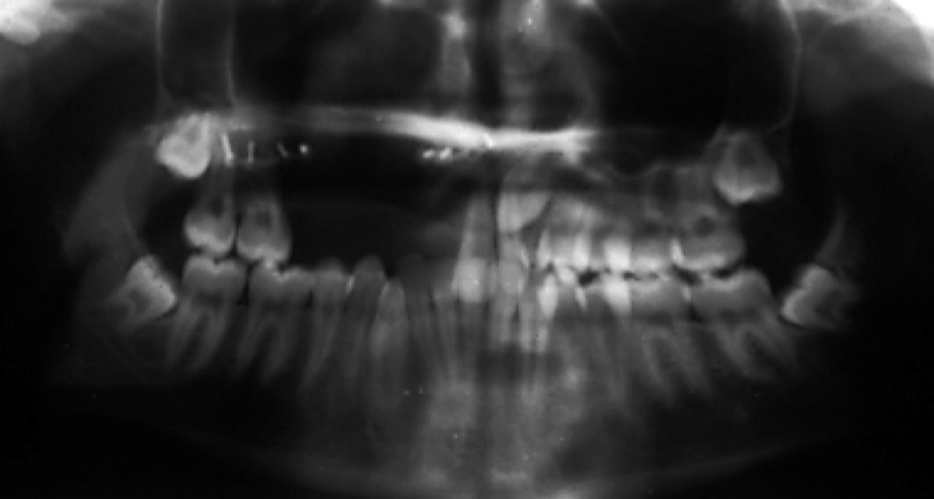
Case report 2. Panoramic X-ray after bone grafts from iliac crests fixed with titanium plates.

After malignant diagnosis, the patient was referred and treated in the Oral and Maxillofacial Service at the Bellvitge University Hospital. General anesthesia was used to perform the partial resection of upper right maxilla from central incisor to second premolar. No neoplastic infiltration was noted. Reconstruction was carried out with autogenous bone grafts obtained from the iliac crests and titanium plates during the same surgical procedure. No postoperative complications were reported (Fig. 6).

After one year, as no recurrence was observed four dental implants (Nobel Speedy Groovy®; Nobel Biocare, Zurich, Switzerland) were placed in order to rehabilitate the edentulous span.

Follow-up visits have been performed every 6 months with no recurrence of the malignant lesion. However, in 2010, one implant failed due to peri-implantitis. Actually, the prosthesis is supported by three successful implants.

## Discussion

The epithelial lining of odontogenic cysts may experience simple cystic expansion, keratinization, mucous prosoplasia, or dysplastic transformation (6). The most common neoplasms arising inodontogenic cysts are benign tumours such as the odontogenic adenomatoid tumour, pleomorphic adenoma, as well as serve as a precursor of an ameloblastoma (7). Although very unlikely, malignant transformation of an odontogenic cyst into a PIOSCC may occur, most of them arise in radicular/residual cysts, followed by dentigerous cysts, keratocysticod ontogenic tumour and lateral periodontal cysts (1,4).

The real pathogenesis of PIOSCC still remains unclear. Nevertheless, it has been suggested that the longstanding chronic inflammation might be the main predisposing factor to induce a malignant transformation in the cyst epithelium (8,9). Bodner *et al.* (1) suggested three main mechanisms by which inflammation can initiate and promote carcinogenesis.

- Chronic inflammation is often accompanied by the formation of reactive oxygen and nitrogen species by phagocytes. These have the potential to damage DNA, proteins, and cell membranes, modulate enzyme activities and gene expression, promoting carcinogenesis. Moreover, chronic inflammation appears to promote apoptosis of normal cells that leads to a compensatory proliferative response by the remaining cells. This process increases the number of cells that are dividing and therefore are subject to DNA damage and promotes the growth of malignant cells.

- Infectious agents may directly transform cells by inserting active oncogenes into the host genome, inhibiting tumour suppressor or stimulating mitoses.

- Infectious agents may induce immunosuppression with consequent reduced immunosurveillance.

Genetic factors have been also suggested to play a role in the carcinogenesis initiation and progression. In this sense, recent studies suggest that there is a relationship between the epidermal growth factor receptor distribution in pericoronal follicles and the origin of odontogenic cysts and tumors (10).

A review of the literature from a total of 30 cases, including our report, on PIOSCC originated from dentigerous cysts was performed in order to summarize the main clinical features, treatment options and prognosis of this entity (5,7,11-35) ( Table 1). Dentigerous cyst malignization into PIOSCC mostly occurs from the 3rd decade of life; the male to female ratio was 2.3:1. Male gender predilection it not has to be considered as to PIOSCC but rather reflects the higher pre-existing occurrence of odontogenic cysts among men (36).

**Table 1:**
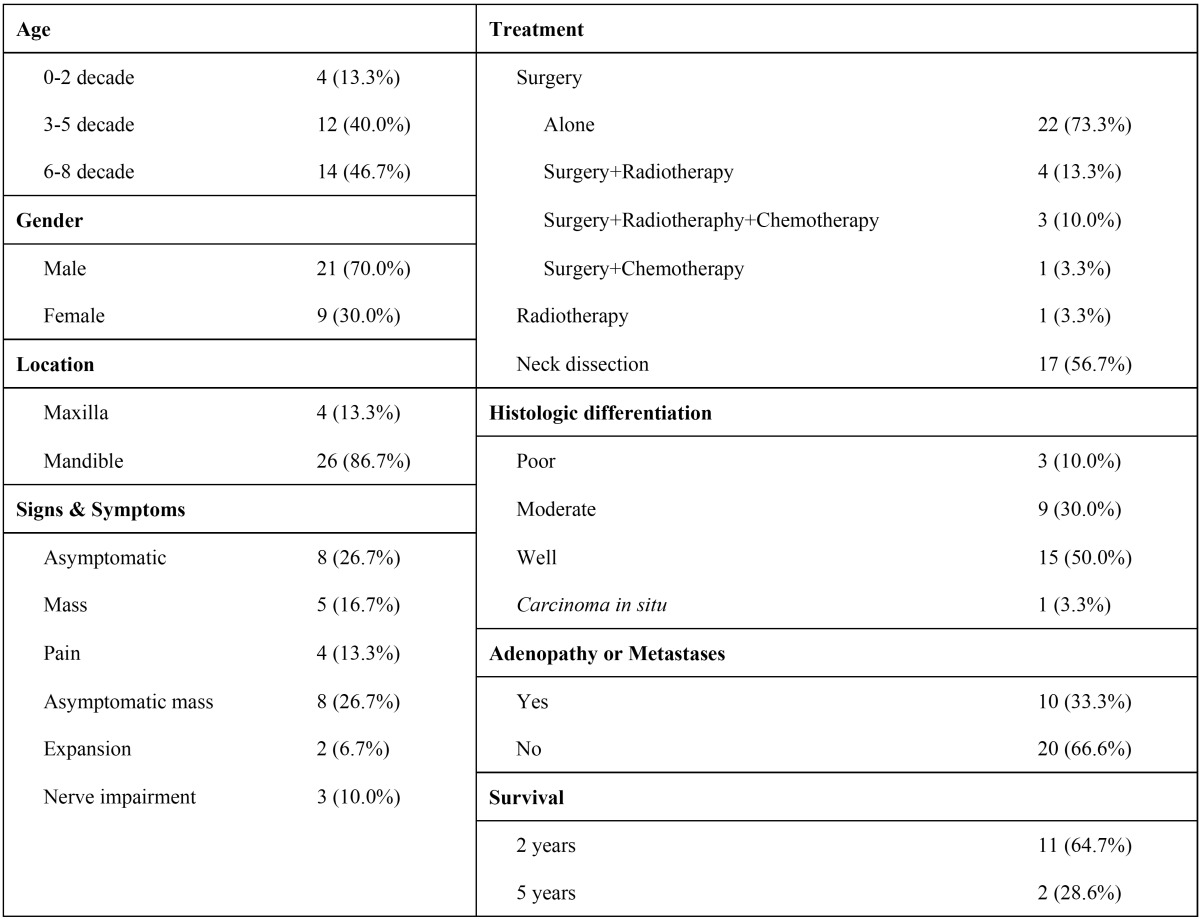
Summary of the main clinical, therapeutic and prognostic features of the 30 cases of PIOSCC arising indentigerous cysts reported (5,7,11-35).

Clinically, according to the typical features of non-infected dentigerous cysts, PIOSCC appears to be painless in the majority of cases (37). Nevertheless, in case of clinical manifestations appearance, swelling was the most frequent sign (43.4%), followed by pain (13.3%) and nerve impairment (10.0%). The mandible was involved in most of the cases (86.7%).

PIOSCC’s radiological features are apparently innocuous; showing a unilocular cystic-like radiolucency, varying in size, shape and margins. Occasionally it may also present a multilocular aspect (38). In other cases, the margins are irregular or poorly defined (10,22).

The deﬁnitive diagnosis of PIOSCC is often difficult and usually made in retrospect due to lack of pathognomonic symptoms and radiographic changes. In any case, this fact forces to perform a routinely accurate anamnesis and physical examination and complementary tests-computed tomography and/or magnetic resonance, chest imagingand biopsy- as may be necessary to ensure the benign nature of the lesion. Moreover, in advanced cases, positron emission tomography is mandatory to assessganglionar involvement, distant metastases, and synchronous second primary tumors (28).

Due its low incidence and the absence of neither clinical nor radiological pathognomonic features; initial treatment is lesion enucleation in all of the cases. Once the histopathological examination is concluded treatment approach for PIOSCC should be determined by the extent of the carcinoma (6). In this sense, a second and more radical treatment may be necessary by means of surgical resection together with radio and/or chemotherapy if the margins of the tumour are positive or there is carcinoma in the surrounding bone (7,22). According to our findings, most of cases required partial hemimandibulectomy or maxillectomy (73.3%) in conjunction with neck dissection (56.7%) to ensure the complete removal of the primary lesion ( Table 1).

Fortunately, neck metastases from PIOSCC are rare (1). Our results are in accordance with these findings since in two-thirds of the cases neither ganglionar nor metastatic affection was reported ( Table 1). Neck metastasis is an important prognostic factor and perhaps affects survival; in such advanced head and neck squamous cell carcinomas immunotherapy is an emerging therapeutic option. In this regard, the monoclonal antibody cetuximabin conjunction with radiotherapy may improve survival rates of recurrent or metastatic cases (39).

The majority of cases of PIOSCC are associated with a well or moderately differentiated squamous cell carcinomas (1). Our results agree with those findings because 80% of the reviewed cases had these histopathologic differentiation ( Table 1).

The overall survival rate of patients with a PIOSCC was 64.7% and 28.6% at 2 and 5 years, respectively ( Table 1).

Squamous cell carcinoma arising in a dentigerous cyst is extremely rare. Due to the fact that malignant transformation occurs unnoticed from both medical and radiological features, complete excision and routine histopathological studies are mandatory. Some patients reporting apparently banal radiographs and symptoms may be carriers of potentially malignant lesions. Future similar cases should be published in order to obtain more information on this rare entity.
